# Enhanced Cellular Immunity for Hepatitis B Virus Vaccine: A Novel Polyinosinic-Polycytidylic Acid-Incorporated Adjuvant Leveraging Cytoplasmic Retinoic Acid-Inducible Gene-Like Receptor Activation and Increased Antigen Uptake

**DOI:** 10.34133/bmr.0096

**Published:** 2024-10-28

**Authors:** Xuhan Liu, Qiuxia Min, Yihui Li, Siyuan Chen

**Affiliations:** ^1^ Guangdong Provincial Key Laboratory of Chinese Medicine Ingredients and Gut Microbiomics, Institute for Inheritance-Based Innovation of Chinese Medicine, Marshall Laboratory of Biomedical Engineering, School of Pharmacy, Shenzhen University Medical School, Shenzhen University, Shenzhen 518055, China.; ^2^ Department of Pharmacy, First People’s Hospital of Yunnan Province, Kunming University of Science and Technology, Kunming, 650034 Yunnan, China.; ^3^ Guangdong Provincial Key Laboratory of Malignant Tumor Epigenetics and Gene Regulation, Guangdong-Hong Kong Joint Laboratory for RNA Medicine, Medical Research Center, Sun Yat-Sen Memorial Hospital, Sun Yat-Sen University, Guangzhou 510120, China.; ^4^ Research Institute for Biomaterials, Tech Institute for Advanced Materials, Bioinspired Biomedical Materials & Devices Center, College of Materials Science and Engineering, Jiangsu Collaborative Innovation Center for Advanced Inorganic Function Composites, Suqian Advanced Materials Industry Technology Innovation Center, Nanjing Tech University, Nanjing 211816 China.

## Abstract

Conventional aluminum adjuvants exhibit limited cellular immunity. Polyinosinic-polycytidylic acid (poly I:C) activates cytoplasmic retinoic acid-inducible gene-like receptor (RLR), triggering strong T cell activation and cellular responses. However, when applied as an adjuvant, its limited endocytosis and restricted cytoplasmic delivery diminish its effectiveness and increase its toxicity. Hybrid polymer–lipid nanoparticle (PLNP) possesses numerous benefits such as good stability, reduced drug leakage, simple fabrication, easy property modulation, and excellent reproducibility compared to the lipid nanoparticle or the polymeric vector. Here, we developed a novel cationic polymer–lipid hybrid adjuvant capable of incorporating poly I:C to enhance cellular immunity. The hepatitis B surface antigen (HBsAg) was immobilized onto poly I:C-incorprated PLNP (PPLNP) via electrostatic interactions, forming the HBsAg/PPLNP vaccine formulation. The PPLNP adjuvant largely enhanced the cellular endocytosis and cytoplasmic transport of poly I:C, activating RLR followed by promoting type I interferon (IFN) secretion. Meanwhile, PPLNP obviously enhanced the antigen uptake, prolonged antigen retention at the site of administration, and facilitated enhanced transport of antigens to lymph nodes. The HBsAg/PPLNP nanovaccine led to amplified concentrations of antigen-specific immunoglobulin G (IgG), IFN-γ, granzyme B, and an enhanced IgG2a/IgG1 ratio, alongside the FasL^+^/CD8^+^ T cell activation, favoring a T helper 1 (T_H_1)-driven immune response. PPLNP, distinguished by its biocompatibility, ease of fabrication, and effectiveness in augmenting cellular immunity, holds significant promise as a new adjuvant.

## Introduction

Vaccination stands as a remarkably efficacious approach in the battle against viral infectious diseases [1,2]. Hepatitis B, a viral ailment with detrimental impacts on the liver, has the potential to induce acute or chronic illnesses. In 2019, it was reported that around 296 million individuals were affected by hepatitis B virus (HBV) infections [3]. Additionally, a remarkable 80% coverage of the full 3-vaccination schedule regarding HBV has been achieved globally [4], and the aluminum salts are most widely used adjuvants in clinical practice [5,6]. These aluminum adjuvants are particularly efficient at boosting humoral immune responses [T helper 2 (T_H_2) type] by adsorbing antigens via electrostatic interactions, and enhance antigen recognition, endocytosis, and presentation [7,8]. However, the aluminum adjuvants have limited efficacy in stimulating cellular immune responses (T_H_1 type), which is suboptimal for viral diseases necessitating T_H_1-mediated immunity and cytotoxic T cell activation for protection [7].

Except for enhancing the antigen uptake, activation of pattern recognition receptors (PRRs) played a greater significance in activating cellular immunity. The cytosol harbors numerous targets [9–11], which reside within intracellular compartments, playing a vital role in enhancing cellular immunity and protecting against intracellular pathogens [12,13]. For example, viruses are usually internalized with the help of spike proteins and uncoated and then deliver their genome to the cytoplasm for replication [14,15]. Upon recognition of genetic RNA, the cytoplasmic retinoic acid-inducible gene-like receptor (RLR) can be activated to mount an antiviral cellular immune response [16]. Therefore, inspired by the process of viral activation of cellular immunity, developing a novel adjuvant that significantly promotes antigen endocytosis and simultaneously activates RLR holds the promise of significantly enhancing cellular immune responses [17].

Polyinosinic-polycytidylic acid (poly I:C), which is a double-stranded RNA (dsRNA) analog, can effectively stimulate T_H_1-type immune responses through interaction with cytoplasmic RLR like retinoic acid-inducible gene I (RIG-I) [18]. However, its effective application faces various challenges, including limited cell uptake due to its diffuse form and negative charge, and off-target effects causing excessive inflammation and tissue damage. Since common carriers are eventually trafficked to lysosomes after internalization into cells, and poly I:C is highly susceptible to degradation in lysosomes, its efficiency of entering the cytoplasm to activate RLRs is low [19]. Hence, a suitable nanocarrier with efficient loading capacity of poly I:C, facilitating its internalization and potent activation of RLRs, is pivotal in designing adjuvants to augment cellular immunity.

**Fig. 1.**
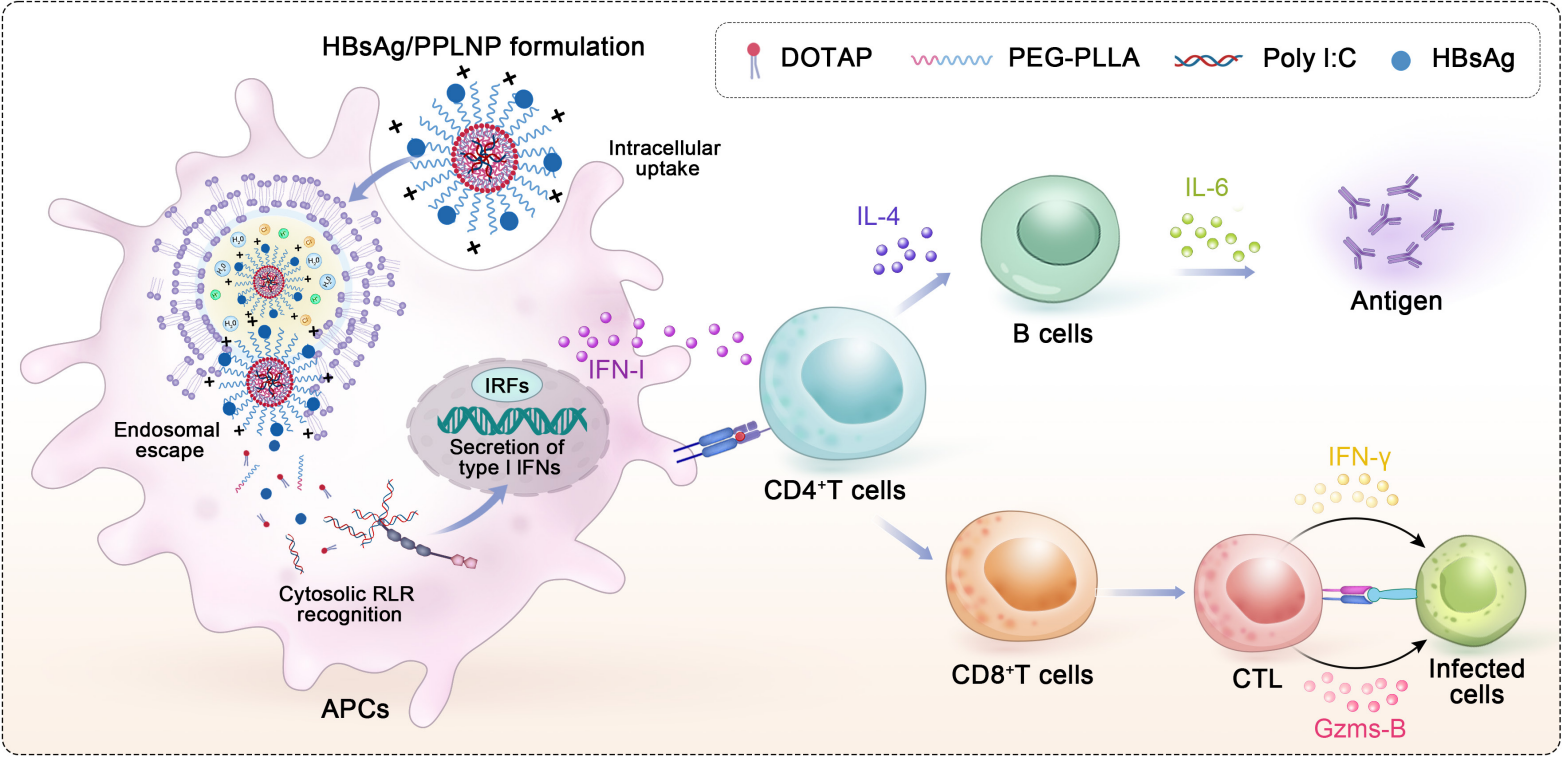
The composition of the HBsAg/PPLNP formulation and its mechanism for enhancing cellular immunity, leveraging cytosolic RLR activation and increased antigen uptake.

Cationic nanoparticles would have the endosomal escape capacity for cytoplasmic delivery due to the “proton sponge effect” produced by positive charge [20]. Although some reported that vectors can achieve cytoplasmic delivery [21], they still have some issues. For example, lipid nanoparticles have a phospholipid bilayer structure that, due to its good fluidity, can easily lead to drug leakage [22]. Some cationic polymers have characteristics of difficult degradation and toxicity, such as polyethyleneimine (PEI), or are synthetically complex with significant batch-to-batch variability, such as chitosan [23]. Hybrid polymer–lipid nanoparticle (PLNP) possesses numerous benefits such as good stability, reduced drug leakage, simple fabrication, easy property modulation, and excellent reproducibility [24,25]. Therefore, we plan to create a straightforward, reproducible hybrid PLNP adjuvant composed of the amphiphilic polymer polyethylene glycol-poly(l-lactic acid) (PEG-PLLA) and the cationic lipid 1,2-dioleoyl-3-trimethylammonium-propane (chloride salt) (DOTAP). Using a straightforward one-step approach, poly I:C could be efficiently encapsulated. Antigen proteins can be finally adsorbed on the surface, forming a vaccine formulation. Functionally, the poly I:C-incorprated PLNP (PPLNP) could efficiently activate cytoplasmic RLRs and enhance the endocytosis of antigens, thereby facilitating cellular immunity (as seen in Fig. 1). Here, we conducted a comprehensive assessment of the properties of PPLNP and investigated the interactions between PPLNP and antigen-presenting cells (APCs), and the in vivo immunological effectiveness of PPLNP. Our findings highlight the distinctive features of PPLNP and underscore its potential as an adjuvant to enhance cellular immunity.

## Materials and Methods

### Materials

Tetrahydrofuran (THF), paraformaldehyde fixation solution, fluorescein isothiocyanate (FITC), 4′,6-diamidino-2-phenylindole (DAPI), LysoTracker Green, Triton X-100, and bovine serum albumin (BSA) were procured from Sigma-Aldrich. DOTAP was bought from Avanti Polar Lipids. mPEG_5k_-PLLA_10k_ was purchased from Tansh Tec Co. Ltd. (China). The Cell Counting Kit-8 (CCK-8) assay was sourced from Abcam (Cambridge, UK). Trypsin-EDTA, fetal bovine serum (FBS), and penicillin-streptomycin were purchased from Gibco (CA, USA). Phosphate-buffered saline (PBS), RPMI 1640 medium, and high-glucose Dulbecco’s modified Eagle’s medium (DMEM) were purchased from HyClone (UT, USA). Fixation buffer and Perm/Wash solution were procured from Nuowei Biotechnology Co. Ltd. (Beijing, China). Sulfo-cyanine5.5 (Cy5.5) and FITC fluorescently labeled antibody/protein kit were obtained from Beijing Zhongkeliyan Biotechnology Co. Ltd. (China). 2-(4-Amidinophenyl)-6-indolecarbamidine dihydrochloride (DAPI) was bought from GuangZhou Sopo Biological Technology Co. Ltd. (China). Antibodies of peridinin chlorophyll protein (PerCP)/Cy5.5-CD3 and ER780-CD8a were sourced from Elabscience. Antibodies including Ms CD4 FITC GK1.5, Ms CD8a PerCP-Cy5.5 (53-6.7), Ms CD19 allophycocyanin-Cy7 1D3, Ms CD69 allophycocyanin H1.2F3, and Ms CD178 phycoerythrin (PE) MFL3 were obtained from Becton, Dickinson and Company (New Jersey, USA). FITC anti-mouse major histocompatibility complex class II (MHCII) (I-A/I-E) antibody was obtained from SALMART (Shenzhen, China). Mouse hepatitis B surface antigen (HBsAg)-specific antibody enzyme-linked immunosorbent assay (ELISA) kits like immunoglobulin G (IgG), IgG1, and IgG2a were acquired from Shanghai Runyu Biotechnology Co. Ltd. (China). Mouse ELISA kits of interleukin-4 (IL-4), IL-6, interferon-γ (IFN-γ), granzyme B (Gzms-B), and RAW264.7 cells were obtained from Beyotime (Shanghai, China). Antibodies of anti-mouse FITC-CD11c, PerCP/Cy5.5 anti-mouse CD80, PE/cyanine7 anti-mouse CD86, and allophycocyanin anti-mouse MHCII (I-A/I-E) were purchased from BioLegend. The mouse renal tubular epithelial cell (TCMK-1), the mouse fibroblast cell (NIH 3T3), and the human liver cancer cell (HepG2) were kindly provided by the center laboratory of Shenzhen University General Hospital. Sheep blood was obtained from Yuchen Biology Co. Ltd. (Shanghai, China). HBsAg was generously gifted by AIM Honesty Biopharmaceutical Co. Ltd. (Dalian, China). Aluminum oxide adjuvant was bought from Miragen (Delaware, USA). Poly I:C (molecular weight = 3,500) was obtained from Shanghai Yuanye Biotechnology Co. Ltd. Rhodamine-labeled poly I:C was obtained from InvivoGen. RiboGreen RNA assay kit was sourced from Thermo Fisher Scientific.

### Preparation and characterization of PPLNP adjuvant

We prepared PPLNPs by a straightforward one-pot method. Briefly, 850 μg of PEG_5k_-PLLA_10k_ and 350 μg of DOTAP were co-dissolved in THF. To prepare the aqueous phase, 50 μg of poly I:C (molecular weight = 3,500) was dissolved in water. The organic phase, containing PEG-PLLA and DOTAP, was added to the aqueous phase with a volume ratio of 1:2 in one swift motion and stirred at approximately 400 rpm for uniform mixing. The mixture was continuously stirred in a well-ventilated cabinet until the THF was completely evaporated, resulting in the formation of PPLNPs [26]. Then, their average size and zeta potentials were assessed by dynamic light scattering (DLS). The control group was prepared by rapidly mixing THF with water without poly I:C, and the resulting PLNP was also characterized by DLS. The encapsulation efficiency (EE) of poly I:C in PPLNP was analyzed by RiboGreen assay kit [27]. Additionally, the morphology of PPLNPs was checked using transmission electron microscopy (TEM).

### Hemolysis of PPLNP

Sheep erythrocytes [red blood cells (RBCs)] underwent centrifugation at 1,500*g* for 10 min at 4 °C, followed by 3 washes with PBS, and then prepared as a 5% (v/v) suspension in PBS buffer. The PPLNP and Al adjuvant were introduced into the RBC suspension at 0.1 mg ml^−1^. The RBC suspension was incubated at 37 °C for 1 h. After centrifugation, the supernatant was obtained and the absorbance (*A*) at 540 nm was determined by microplate reader (Promega, USA). The hemolysis of samples was analyzed based on the below formula [28]:

$$\text{Relative hemolysis}\;\left(\%\right)=\frac{A_{S}\;-\;A_{\mathrm{NC}}}{A_{\mathrm{PC}}\;-\;A_{\mathrm{NC}}}$$

The subscripts S, NC, and PC denote sample, negative control (treated with PBS), and positive control (treated with deionized water), respectively.

### Endocytosis and cytoplasmic delivery of poly I:C by PPLNP

To assess the endocytosis and intracellular release of poly I:C, 2 × 10^5^ mouse mononuclear macrophages RAW264.7 were seeded in a confocal culture dish for 24 h. Subsequently, the existing medium was exchanged for a fresh serum-free medium, and poly I:C or PPLNP was added at 1 μg ml^−1^ of poly I:C following another incubation for 4 h. After PBS washing, the cells were dyed with DAPI and LysoTracker Green for 10 min. Following fixation with paraformaldehyde (4%), the cells were analyzed with a confocal laser scanning microscope.

Rhodamine-labeled poly I:C was chosen for studying the endocytosis and intracellular release of poly I:C [29]. Similarly, 6 × 10^5^ RAW264.7 cells were placed in a 12-well plate and incubated for 24 h. After substituting with new FBS-free medium, poly I:C or PPLNP was added at 0.5 μg ml^−1^ of poly I:C for 6 h. After rinsing with PBS buffer, the percentage of rhodamine-positive cells were tested using flow cytometry. Meanwhile, RAW264.7 cells were similarly incubated with poly I:C or PPLNP at 0.5 μg ml^−1^ of poly I:C for 24 h, dyed with FITC MHCII antibody, and subsequently analyzed for the MHCII expression using flow cytometry.

### RLR activation and type I IFN secretion

Human embryonic kidney HEK293 cells were cultured in complete high-glucose DMEM with 5% CO_2_ at 37 °C. HEK293 cells (3 × 10^5^) were placed in a 6-well plate and incubated for 18 h. Plasmid DNA (consisting of 2 μg of RIG-I, 2 μg of interferon stimulated response element luciferase reporter plasmid, and 0.2 μg of internal reference) was dissolved in 250 μl of serum-free medium. EZ Trans transfection reagent (20 μl) was dissolved in 250 μl of serum-free medium and quickly added to the diluted plasmid DNA and gently mixed well. The mixture was allowed to incubate for about 10 to 20 min to facilitate the formation of the EZ Trans-DNA complex. Then, the original culture medium in the 6-well plate was discarded, and 1 ml of complete medium was added. The 500-μl EZ Trans-DNA transfection complex was then added to each well and gently mixed well. After 24 h of transfection (from the time of adding the transfection complex), 1 × 10^4^ cells were trypsinized, counted, and replated in a 96-well plate. When the cells were ready, poly I:C or PPLNP formulations were introduced to achieve 0.5, 0.25, 0.125, 0.063, 0.032, 0.016, and 0.008 μg ml^−1^ of poly I:C for another 24 h. Finally, the relative luciferase unit was analyzed with the help of plate reader using a luciferase assay kit [30].

RAW264.7 cells (1 × 10^4^) were placed in a 96-well plate for 24 h. Poly I:C or PPLNP was then introduced at 1 μg ml^−1^ of poly I:C for 48 h, with PBS used as the control. The supernatant was collected for analysis of secreted IFN-α and IFN-β using ELISA kit.

### Preparation and characterization of HBsAg/PPLNP formulation

The HBsAg/PPLNP vaccine was obtained by simply mixing with HBsAg and PPLNP (w/w = 1:625), followed by an incubation at 4 °C for 30 min to facilitate stable formulation via electrostatic adsorption. Unless otherwise specified, the HBsAg/PPLNP formulation mentioned below was maintained at this fixed HBsAg to PPLNP mass ratio. Similarly, its average size and zeta potential were analyzed using DLS, and its morphology was observed using TEM. The positive HBsAg/Al control group was obtained by combining HBsAg and commercial Al adjuvant (w/w = 1:25), according to the instructions.

### In vitro antigen release

The Cy5.5-conjugated BSA (Cy5.5-BSA) was prepared utilizing Cy5.5 fluorescent labeling antibody/protein kit [31]. Subsequently, the BSA/PPLNP formulation was formulated with a weight ratio of 1:625 through a straightforward mixing process of Cy5.5-BSA and PPLNP for in vitro antigen release study. The BSA, BSA/Al (w/w = 1:25), and BSA/PPLNP formulations were incubated in a dialysis bag with molecular weight cutoff of 100 kDa in PBS at 37 °C for 48 h. To assess the in vitro antigen release, the fluorescence intensity of the release medium was measured at designated time intervals using a fluorescence spectrophotometer.

### Cell viability

RAW264.7 cells were grown in complete high-glucose DMEM with 5% CO_2_ at 37 °C. Cell viability in the presence of HBsAg/PPLNP was evaluated. Cells (5 × 10^4^) were plated in a 96-well plate for 24 h. Following this, the medium was replaced with the serum-free medium with different concentrations of HBsAg/Al or HBsAg/PPLNP formulation, and the cells were incubated for 24 h. Subsequently, 10 μl of CCK8 solution was introduced for 4 h, and the absorbance (at 450 nm) was then tested. Additionally, the cytotoxicity of HBsAg/PPLNP was evaluated on other cell lines, including TCMK-1, NIH 3T3, and HepG2.

### Antigen uptake

FITC-conjugated HBsAg was obtained using the respective fluorescent labeling antibody kit. Briefly, 2 × 10^5^ RAW264.7 cells were placed in a confocal dish and incubated for 24 h. Cells were then treated with HBsAg, HBsAg/poly I:C (prepared by simply mixing HBsAg and free poly I:C at a mass ratio of 1:25), or HBsAg/PPLNP (w/w = 1:625) for 4 h, with the concentration of HBsAg kept at 1 μg ml^−1^. After fixation with 4% paraformaldehyde, cells were washed with PBS and stained with DAPI. Finally, the endocytosis of antigen in various formulations was analyzed using a fluorescence confocal microscope. To quantitively analyze the antigen uptake, 6 × 10^5^ RAW264.7 cells were placed in a 12-well plate and treated with HBsAg, HBsAg/poly I:C, or the HBsAg/PPLNP formulation for 4 h, maintaining the concentration of HBsAg kept at 1 μg ml^−1^. The percentage of FITC-positive cells was finally analyzed by flow cytometry.

### Animals

Female C57BL/6 mice, aged 6 to 8 weeks, were obtained from Beijing Biotechnology Co. Ltd. These mice were housed in a specific pathogen-free (SPF) environment at the Key Lab of Shen Zhen University General Hospital. All animal experiments strictly adhered to the Regulations for the Care and Use of Laboratory Animals and the Ethical Review Guidelines for Animal Welfare in China (GB/T 35892-2018).

### Antigen depot and lymph node drainage by PPLNP

HBsAg was labeled with Cy5.5 using respective fluorescent labeling kit. Mice were grouped (*n* = 4) and intramuscularly administered with HBsAg, HBsAg/Al, or HBsAg/PPLNP on the right hind leg at 1 μg of HBsAg. Images of the mice after injection were captured at various time intervals using the in vivo FX Pro (Kodak) imaging system. The mean fluorescence intensities (MFIs) of the injected position and the mesenteric lymph nodes were assessed. For the evaluation of dendritic cell (DC) recruitment and maturation, C57BL/6 mice were intramuscularly injected with HBsAg, HBsAg/Al, or HBsAg/PPLNP into the right hind leg at a dosage of 1 μg of HBsAg. After 24 h, the muscle tissue from the injected site was obtained and enzymatically digested to isolate lymphocytes. Splenocytes were then stained with CD11C antibody. Concurrently, mesenteric lymph nodes were harvested and mechanically dissociated to extract lymphocytes, which were subsequently stained with specific antibodies including CD11C, MHCII, CD80, and CD86. After washing and resuspension in PBS buffer, the lymphocytes were examined using flow cytometry.

### HBsAg-specific antibody and cytokine secretion

Female C57BL/6 mice were assigned to PBS, HBsAg, HBsAg/Al, and HBsAg/PPLNP groups (*n* = 6). Each mouse was immunized with the formulation at the dose of 1 μg HBsAg per leg. Mice were administered with booster injections on days 14 and 28, respectively. The mouse body weights were regularly examined. Blood were obtained from the orbital venous plexus of the mice on days 14, 21, 28, 35, and 42. Serum was obtained following centrifugation. The concentrations of serum HBsAg-specific IgG were analyzed using ELISA kits. Additionally, the serum HBsAg-specific IgG1, IgG2a, and serum IL-4, IL-6, IFN-γ, and Gzms-B concentrations on day 42 were determined.

### IFN-γ secretion and splenocyte proliferation

The splenocytes were harvested from the mice, washed, and incubated with complete RPMI 1640 medium. Polyvinylidene difluoride plates were coated with antibodies for 24 h at 4 °C. After washing completely, 200 μl of complete medium was introduced for 30 min. Next, 5 × 10^5^ splenocytes, along with HBsAg at 5 μg ml^−1^, were incubated for 24 h. The plates underwent a second washing, followed by the introduction of a detection antibody for 2 h. After further washing, plates streptavidin-alkaline phosphatase was applied for 1 h. Then, the substrate (bromochloroindolyl phosphate–nitro blue tetrazolium) was introduced to develop the spots in a dark environment. The spots were finally observed with the ChampSpot Elispot II system.

Splenocytes (5 × 10^5^) were placed in a 96-well plate, with some wells receiving 5 μg ml^−1^ HBsAg stimulation and others remaining unstimulated. After 24 or 48 h, cell viability was analyzed using the CCK8 assay kit. The proliferation of splenocyte was analyzed by calculating the ratio of the absorbance of stimulated cells to that of unstimulated cells.

### Splenocyte activation

The splenocytes were harvested and restimulated with HBsAg at a concentration of 5 μg ml^−1^ for 48 h. Then, the splenocytes were washed and subsequently stained with specific antibodies including Ms CD4 FITC GK1.5, Ms CD8a PerCP-Cy5.5 (53-6.7), Ms CD19 allophycocyanin-Cy7 1D3, Ms CD69 allophycocyanin H1.2F3, and Ms CD178 PE MFL3. After washing and resuspension in PBS, the splenocytes were analyzed using flow cytometric analysis.

### Immunohistochemical evaluation

After the initial immunization for 24 h, mice were euthanized. Muscles at the injected part and vital organs were obtained and processed for fixation, followed by embedding in paraffin wax and staining with hematoxylin and eosin (H&E). The stained tissue was finally imaged with a microscope.

## Results

### Preparation and characterization of PPLNP adjuvant

**Fig. 2.**
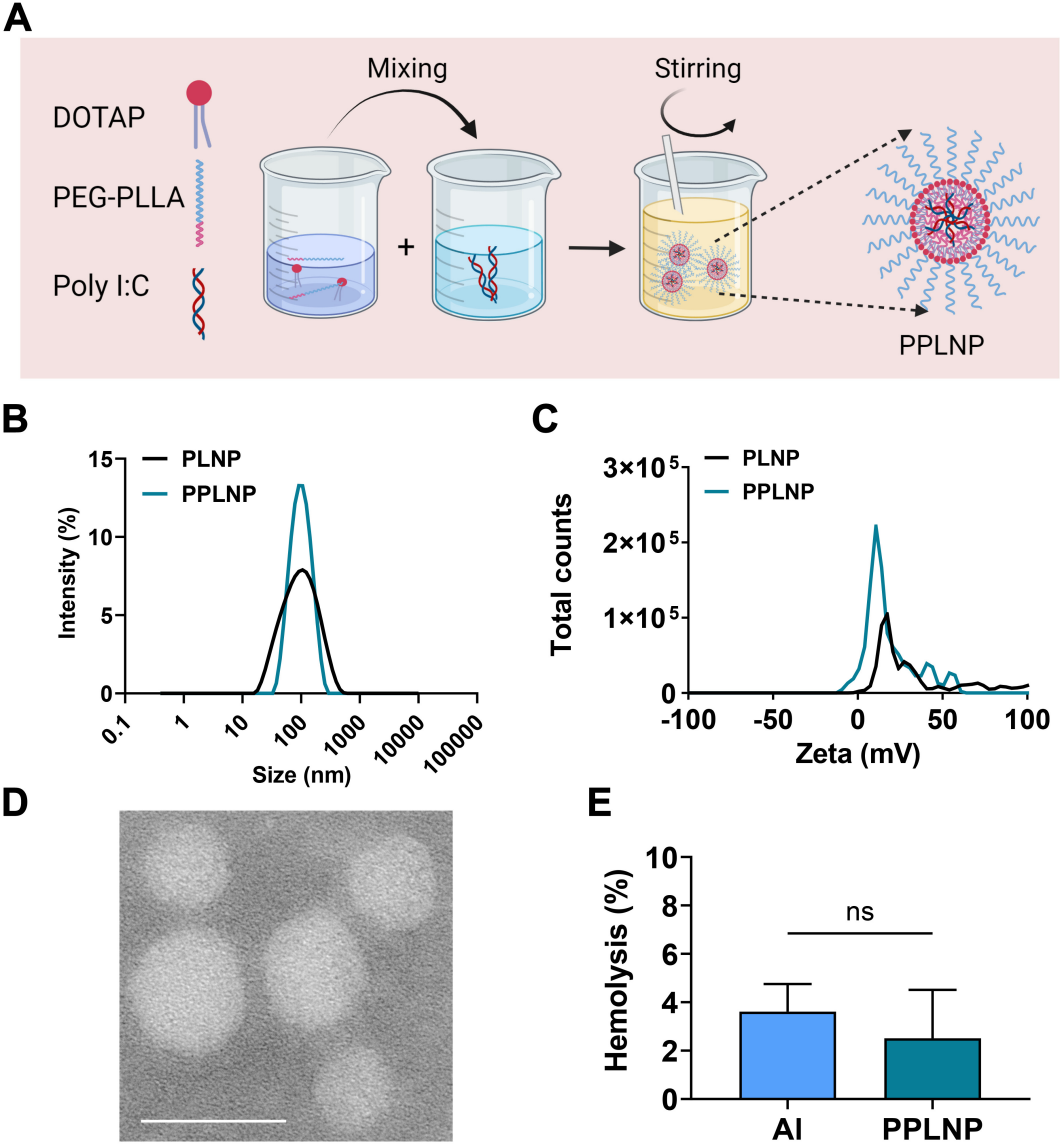
(A) Diagrammatic representation of the fabrication and architecture of the PPLNP adjuvant. Created by BioRender. (B) Average size and (C) zeta potential of PLNP and PPLNP formulations. (D) Morphology of PPLNP observed by TEM. Scale bar, 100 nm. (E) Hemolysis of Al adjuvant and PPLNP at 0.1 mg ml^−1^. Student’s *t* test was chosen for statistical analysis.

PPLNP was prepared using a one-step approach involving mixing, stirring, and evaporation of organic solvent. Poly I:C is a negatively charged, water-soluble analog of dsRNA. PEG-PLLA is an amphiphilic polymer, with the PEG segment being hydrophilic and the PLLA segment hydrophobic. DOTAP is a positively charged, hydrophobic lipid. Since THF is miscible with water, DOTAP can electrostatically interact with poly I:C in the continuously stirred mixture. As THF evaporates, the hydrophobic PLLA segments can interact with DOTAP through hydrophobic interactions, inducing self-assembly. Consequently, poly I:C would be efficiently encapsulated into the PLNP through electrostatic interactions with DOTAP and hydrophobic interactions between DOTAP and PEG-PLLA, forming the structurally stable PPLNP nanoparticle. The composition and structure of the PPLNP vaccine formulation was illustrated (as seen in Fig. 2A). PPLNP exhibited an average diameter of 83.9 ± 0.3 nm, and the polydispersity index (PDI) of PPLNP was 0.27 ± 0.03 (Fig. 2B). Additionally, PPLNP exhibited a positive surface charge of approximately 19.1 ± 2.3 mV (Fig. 2C). The size distribution and PDI of PLNP were approximately 73.0 ± 1.5 nm and 0.32 ± 0.034, respectively. The zeta potential of PLNP was approximately 40.9 ± 1.3 mV. The results indicated that after loading poly I:C, the size distribution of PPLNP increased from 73.0 nm to approximately 83.9 nm, while the zeta potential decreased from 40.9 mV to 19.1 mV. TEM images (as seen in Fig. 2D) illustrated the morphology of PPLNP. The TEM data revealed a slightly smaller size distribution compared to the DLS data. This discrepancy arises because DLS analysis, performed on particles in suspension, can be influenced by aggregation and solvent effects, whereas TEM directly images the physical diameter of the dried particles. The EE of poly I:C in PPLNP was about 82.5 ± 0.8%, tested by RiboGreen assay kit. PPLNPs exhibited minimal hemolytic activity, registering at less than 5% (as seen in Fig. 2E).

### Endocytosis and intracellular release of poly I:C

**Fig. 3.**
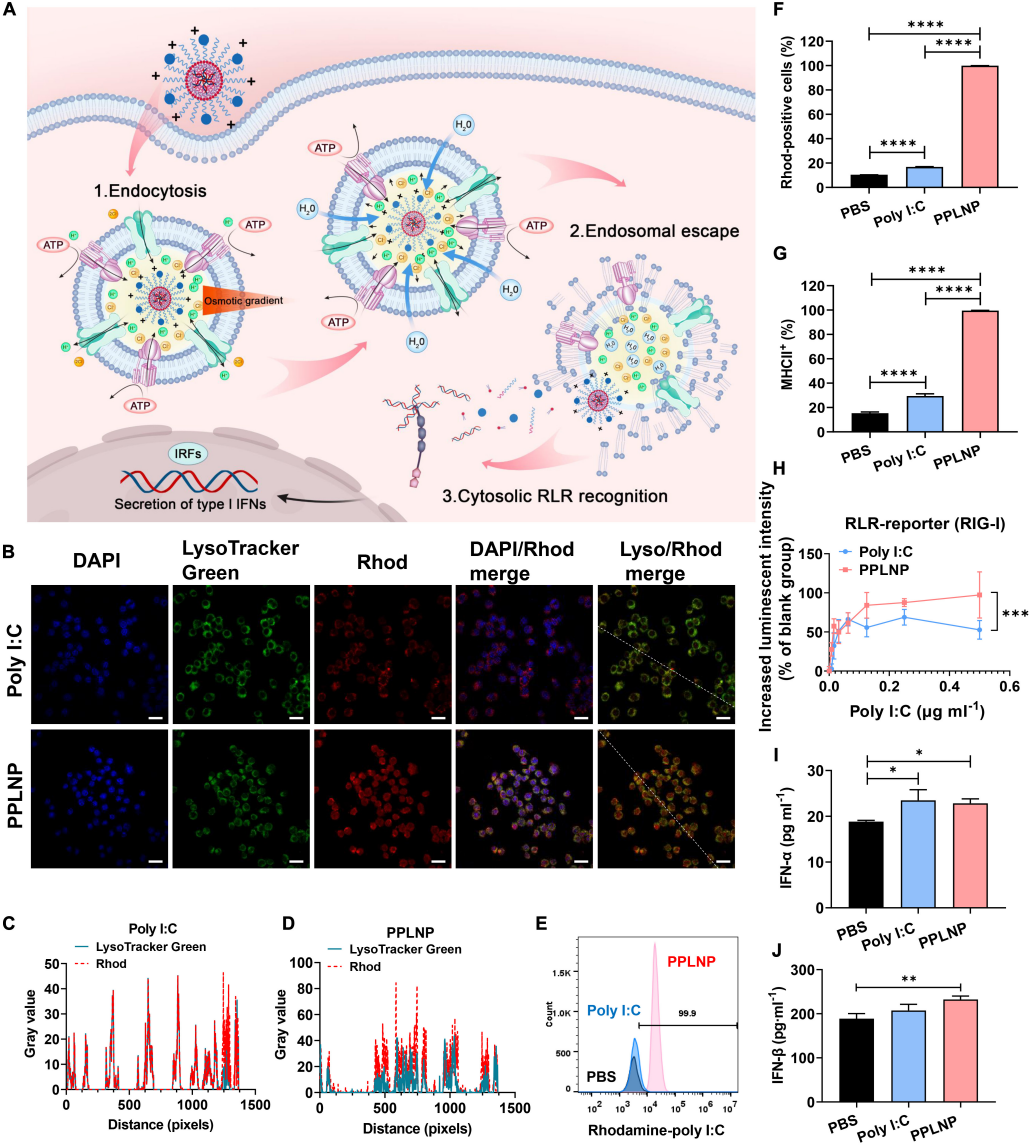
(A) Potential diverse immune-enhancing effects with the HBsAg/PPLNP vaccine formulation by stimulating RLR. (B) Intracellular uptake and release of poly I:C after incubation with poly I:C or PPLNP (1 μg ml^−1^ of poly I:C) for 4 h by confocal microscope. Scale bar, 20 μm. (C) Cellular LysoTracker Green and rhodamine (Rhod) colocalization after treatment with poly I:C by grayscale intensity variations. (D) Cellular LysoTracker Green and rhodamine colocalization after treatment with PPLNP by grayscale intensity variations. (E) Flow cytometry graph and (F) percentage of rhodamine-positive RAW264.7 cells after incubation of poly I:C or PPLNP at 0.5 μg ml^−1^ of poly I:C for 4 h. (G) MHCII^+^ cells after exposure to poly I:C or PPLNP for 24 h. (H) Relative increase in luminescent intensity upon RIG-I activation. (I) Concentration of IFN-α and (J) IFN-β in the medium following 48-h incubation of RAW264.7 cells with either poly I:C or PPLNP. *P < 0.05, **P < 0.01, ***P < 0.001, ****P < 0.0001. One-way analysis of variance (ANOVA) or two-way ANOVA analysis was selected.

Due to its negative charge and good solubility, the cellular uptake of poly I:C would be challenging. However, PPLNP would assist the internalization of poly I:C and its cytoplasmic delivery through the “proton sponge effect”. The intracellular journey of most carriers, particularly the endolysosomal pathway, is a key bottleneck that determines the effectiveness of cytoplasmic delivery. As PPLNP was positive charged, it would have the “proton sponge effect” [32], which leads to endosomal rupture through osmotic swelling and poly I:C release in cytoplasm, stimulating RLRs. Specifically, the positive-charged PPLNP is transported into the endolysosomes, where the acidic environment leads to its increased protonation due to its inherent buffering ability. The membrane-bound V-ATPase (adenosine triphosphatase) actively pumps chloride ions into the endolysosomes, creating an osmotic imbalance. To restore equilibrium, water flows into the compartment, causing it to swell. This swelling eventually results in the disruption of the endolysosomal membrane, releasing the cargo into the cytoplasm (as seen in Fig. 3A). Confocal microscopy was employed to observe the internalization and intracellular release dynamics of poly I:C. As illustrated in Fig. 3B, at 4 h after incubation, the intracellular rhodamine fluorescence intensity of the cells treated with PPLNP was notably higher compared to that treated with poly I:C. For a clearer comparison of the lysosome colocalization, the quantitative analysis was carried out. We employed ImageJ software to construct stacks of LysoTracker Green and rhodamine images. A random line was drawn across the image to intersect as many cells as possible (as seen in Fig. 3B). By analyzing the grayscale intensity variations of LysoTracker Green and rhodamine along this line, the colocalization of LysoTracker Green and rhodamine was demonstrated. Figure 3C shows that the LysoTracker Green and rhodamine lines in the poly I:C group were highly overlapped, indicating a high degree of colocalization. This suggested that the free poly I:C was almost entirely retained within the lysosomes. In contrast, the LysoTracker Green and rhodamine lines in the PPLNP group exhibited significant nonoverlap, indicating that the majority of PPLNP (poly I:C) had successfully breached the lysosomal barrier and entered the cytoplasm (as seen in Fig. 3D). Figure 3E depicts a graph illustrating a notable disparity in rhodamine fluorescence between the PPLNP and poly I:C groups. A quantitative analysis of the rhodamine-positive cell ratio revealed that macrophages incubated with the PPLNP group displayed a rhodamine-positive cell ratio approaching 100% (as seen in Fig. 3F), markedly surpassing poly I:C (****P < 0.0001). As depicted in Fig. 3G, only a modest 30% of the cells activated by poly I:C exhibited MHCII expression, in stark contrast to the substantial up-regulation, approaching 100%, of MHCII molecule expression observed in macrophages stimulated by PPLNP (****P < 0.0001).

### RLR activation and type I IFN secretion

In Fig. 3H, it is evident that the intracellular cytoplasmic RLR activation level of the PPLNP group is obviously greater than the free poly I:C (***P < 0.001). It showed the RAW264.7 cells activated by poly I:C or PPLNP can produce obviously higher IFN-α compared to the PBS group (Fig. 3I). Moreover, PPLNP stimulated RAW264.7 cells to secrete higher levels of IFN-β than poly I:C (Fig. 3J).

### Preparation and characterization of HBsAg/PPLNP formulation

Upon mixing with HBsAg antigen, HBsAg/PPLNP with a mass ratio of 1:625 showed an average size of about 67.8 ± 0.7 nm. HBsAg/PPLNP also exhibited a positive charge of approximately 19.4 ± 1.5 mV (Table S1). The TEM graph of the HBsAg/PPLNP formulation was shown in Fig. S1. These results indicated that the physicochemical properties of the HBsAg/PPLNP formulation showed negligible change compared to PPLNP.

### In vitro antigen release

**Fig. 4.**
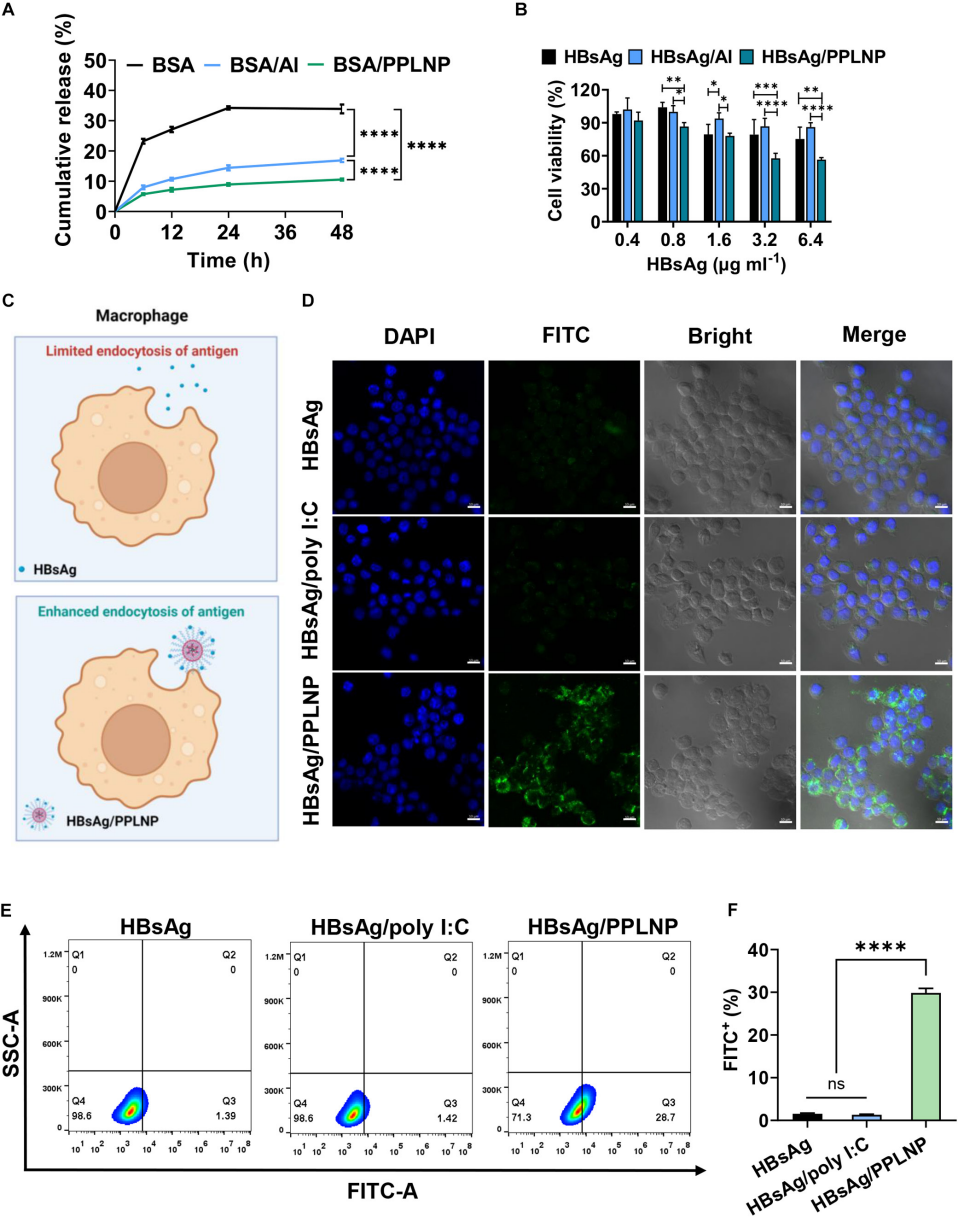
(A) In vitro release of model antigen from various groups in PBS buffer for 48 h. (B) Cytotoxicity of HBsAg, HBsAg/Al, and HBsAg/PPLNP at varying concentrations on RAW264.7 cells after 24 h. (C) Schematic illustration of the endocytosis of HBsAg antigen without/with the PPLNP adjuvant by macrophages. Created by BioRender. (D) Intracellular uptake of HBsAg after incubation with various groups including HBsAg, HBsAg/poly I:C, or HBsAg/PPLNP (1 μg ml^−1^ of HBsAg) for 4 h. Scale bar, 10 μm. (E) Graph of endocytosis of HBsAg in RAW264.7 cells and (F) FITC-positive RAW264.7 cells after exposure to different samples for 4 h. *P < 0.05, **P < 0.01, ***P < 0.001, ****P < 0.0001. We chose one-way ANOVA or two-way ANOVA analysis methods.

BSA, a negatively charged protein commonly employed for release studies, serves as a simulated antigen (Fig. 4A) [31]. In the absence of adjuvants, the cumulative release of BSA steadily increased, reaching a stable value of 34.0% after 24 h. Conversely, the BSA/Al and BSA/PPLNP groups exhibited significantly slower rates of antigen release, not reaching equilibrium until 48 h. Besides, the peak accumulated released antigen of the BSA/Al formulation (approximately 17.2%) and the BSA/PPLNP formulation (approximately 10.6%) was notably lower than in the free antigen group (****P < 0.0001 for both) after 48 h. Importantly, compared to the Al adjuvant, the release of model antigen in formulation adjuvanted with PPLNP was significantly lower (****P < 0.0001).

### Cell viability

Good biosafety is essential for the application of adjuvants, as many adjuvants are constrained for further use by their inherent toxicity. Therefore, our initial assessments focused on testing for cytotoxicity and hemolytic properties, which were commonly linked to cationic adjuvants. As depicted in Fig. 4B, the viability of cells decreased in response to escalating concentrations of the HBsAg/PPLNP formulation. This decline in viability can be ascribed to the positive charge of PPLNPs. However, the cytotoxicity of HBsAg/PPLNP remained within acceptable thresholds, with viability reaching nearly 60% even at higher concentration of 3.2 μg ml^−1^ HBsAg. Importantly, this observation underscores the acceptable cytotoxic profile and favorable biocompatibility of PPLNPs. These results are promising and suggest that PPLNPs warrant further consideration for in vivo investigations. The cell viability of HepG2, NIH 3T3, or TCMK-1 cell lines after being treated with various concentrations of HBsAg/PPLNP was also shown (Fig. S2).

### Elevated cellular uptake of antigen by macrophage

Due to its negatively charged, protein-based macromolecular nature, the HBsAg antigen exhibits limited cellular uptake. In contrast, the positively charged HBsAg/PPLNP formulation significantly enhances antigen uptake, primarily attributed from electrostatic forces between the formulation and the negative-charged cell membrane (as seen in Fig. 4C). As seen in Fig. 4D, the intracellular fluorescence intensity of the HBsAg/PPLNP group was greater than the HBsAg or HBsAg/poly I:C group. Also, the percentage of FITC-positive macrophages in the HBsAg/PPLNP group was around 29.8%, markedly surpassing the HBsAg (~1.6%, ****P < 0.0001) and HBsAg/PPLNP (~1.3%, ****P < 0.0001) groups detected by flow cytometry (as seen in Fig. 4E and F). These results provide compelling evidence that the PPLNP adjuvant significantly enhances the endocytosis of HBsAg compared to the simple mixture of HBsAg and poly I:C. Along with the endocytosis and intracellular release data of poly I:C (Fig. 3), it is clear that PPLNP is also superior in facilitating the internalization of antigen. Consequently, the subsequent in vivo immunization evaluation will exclude the HBsAg/poly I:C group.

### Intensified antigen depot and facilitated lymph node drainage

**Fig. 5.**
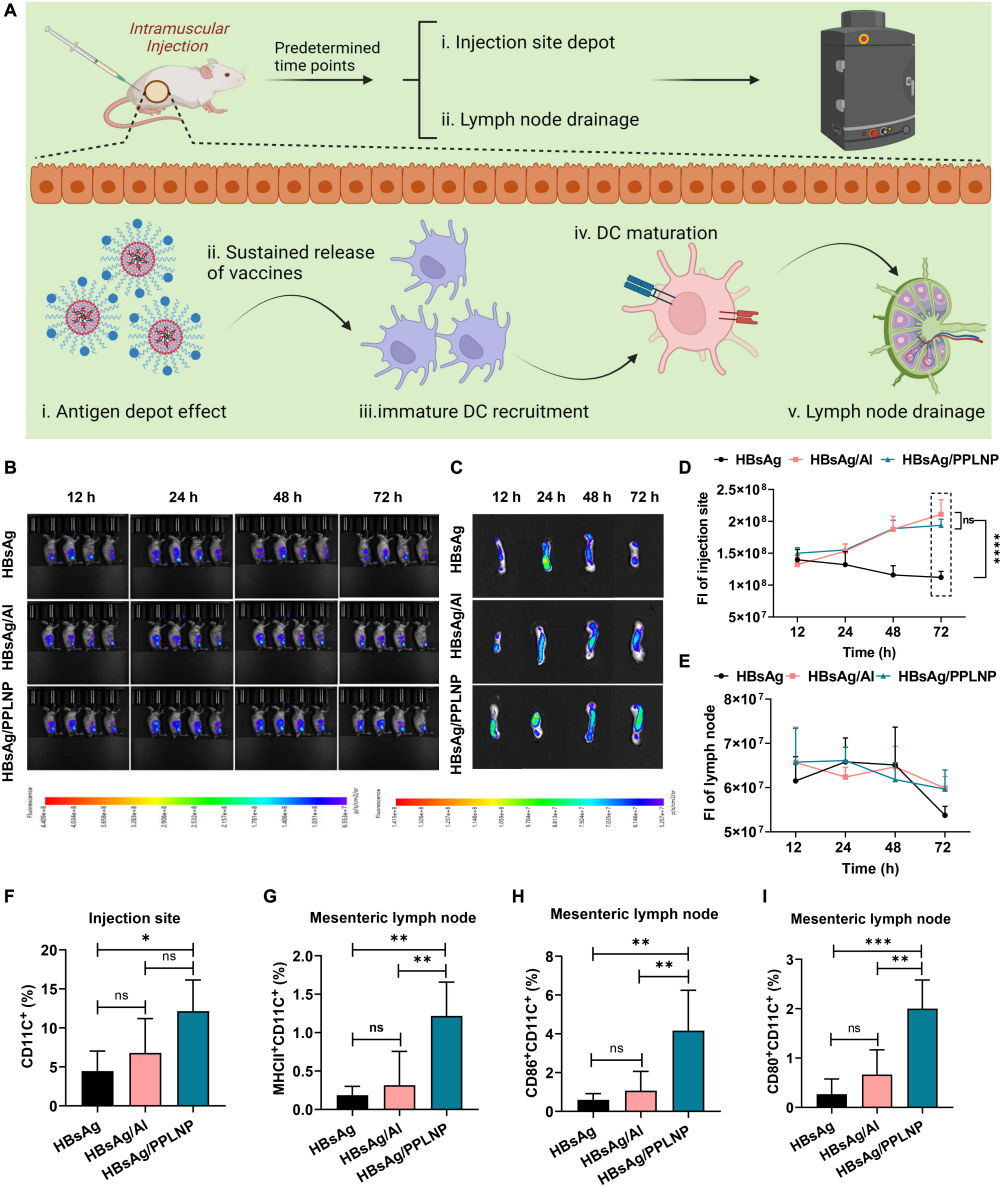
(A) Antigen residency and lymph node drainage. Created by BioRender. (B) Fluorescence images of the injected portions and (C) the mesenteric lymph nodes in mice. (D) Relative FI of the injected portion following the administration of various formulations (1 μg of HBsAg per mouse) during 72 h (****P < 0.0001, HBsAg group versus HBsAg/Al group or versus HBsAg/PPLNP group at 72 h). (E) Fluorescence intensity of mesenteric lymph nodes after being injected with various vaccine formulations at 1 μg of HBsAg per mouse during 72 h. (F) Percentages of CD11C^+^ lymphocytes at injection sites and (G to I) MHCII^+^, CD86^+^, and CD80^+^ cells within CD11C^+^ in mesenteric lymph nodes were assessed 24 h after immunization with various vaccine formulations (1 μg of HBsAg per leg). *P < 0.05, **P < 0.01, ***P < 0.001. Statistical analyses were conducted using either one-way ANOVA or two-way ANOVA methods.

Mice were administered various formulations to evaluate antigen depot formation and lymphatic drainage (Fig. 5A). The obtained images showing varying rates of antigen retention at the injection sites were shown in Fig. 5B. The fluorescence intensity (FI) of the HBsAg group in the injection portion gradually diminished from 1.40 × 10^8^ to 1.12 × 10^8^ within 72 h. However, the FI of the injection portion in the HBsAg/PPLNP group gradually increased (from 1.50 × 10^8^ to 1.94 × 10^8^) along with time, which was similar to the Al adjuvant. Hence, the FI of the injection portion in the HBsAg/PPLNP group after 72 h surpassed that observed at 12 h, and notably exceeded the intensity of the HBsAg group (as depicted in Fig. 5D) (****P < 0.0001). This phenomenon occurred due to the ability of PPLNP to absorb the antigen, thereby prolonging its retention at the injected sites and slowing down the antigen release.

The MFI of the lymph nodes in all these 3 groups was comparable after injection for 48 h, indicating an efficient antigen transportation to mesenteric lymph nodes (Fig. 5C and E). Despite that the FI of the lymph nodes diminished at 72 h, the fluorescence in the HBsAg/Al or HBsAg/PPLNP group was still substantially greater than that observed in the HBsAg group.

The results of DC recruitment and maturation analysis are presented (as seen in Fig. 5F to I). The data indicated that the percentage of CD11C^+^ cells at the injected portion of the HBsAg/PPLNP group was greater than that of the HBsAg group, while negligible difference can be seen in comparison with the Al adjuvant (Fig. 5F). Regarding DC maturation in lymph nodes, the percentage of MHCII^+^, CD86^+^, and CD80^+^ cells within the CD11C^+^ population was markedly greater in the HBsAg/PPLNP group than in the groups without adjuvant or with aluminum adjuvant (as seen in Fig. 5G to I). These suggest that PPLNP effectively enhances the DC recruitment and maturation compared to the Al adjuvant.

### Elevated HBsAg-specific IgG and IgG2a/IgG1 trend

**Fig. 6.**
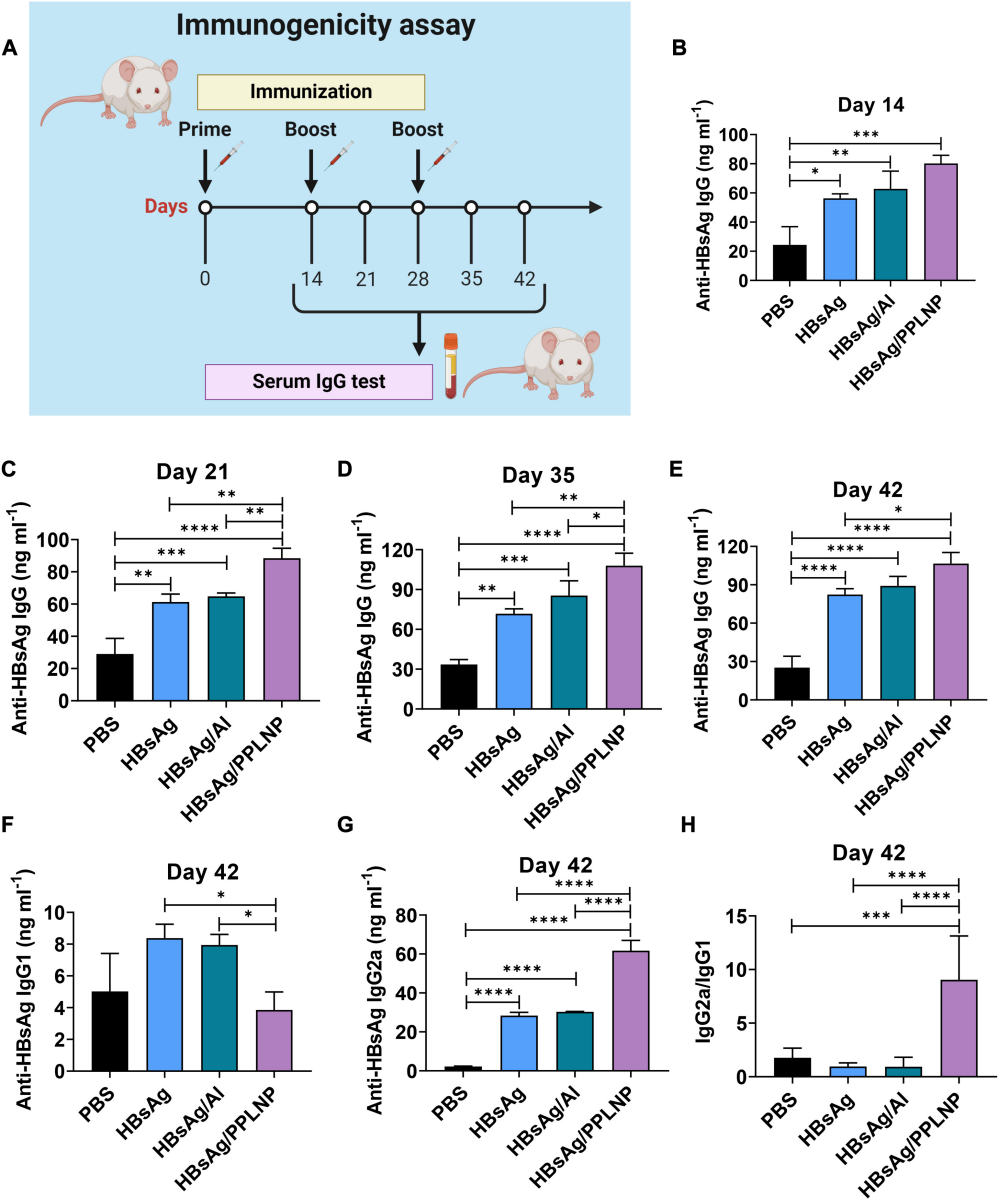
(A) Illustrative schedule depicting the in vivo immunization process and subsequent HBsAg-specific IgG testing. Created by BioRender. (B) Levels of HBsAg-specific IgG after initial immunization with various vaccine formulations at 1 μg of HBsAg per leg on day 14. (C) Concentrations of HBsAg-specific IgG following booster doses with various vaccine formulations at 1 μg of HBsAg per leg on day 21 in serum. (D) HBsAg-specific IgG content after the third injection with various vaccine groups on either day 35 or (E) day 42. (F) Levels of HBsAg-specific IgG1 and (G) IgG2a in serum after booster administration with various vaccine formulations on day 42. (H) Calculation of the IgG2a/IgG1 ratio following booster doses with various vaccine formulations on day 42. *P < 0.05, **P < 0.01, ***P < 0.001, ****P < 0.0001. One-way ANOVA was selected as the statistical analysis method.

Mice were immunized based on the illustration (as seen in Fig. 6A). The anti-HBsAg IgG concentrations in serum at predetermined time points were analyzed. On day 14, all groups receiving HBsAg (alone, with aluminum adjuvant, or with PPLNP adjuvant) showed substantially higher concentrations of HBsAg-specific IgG than did the PBS group after the initial immunization (as seen in Fig. 6B). Following the second injection on day 21, the HBsAg/PPLNP group exhibited a substantially greater level of HBsAg-specific IgG than did the HBsAg group without adjuvant (**P < 0.01) or the group adjuvanted with Al (*P < 0.05) (Fig. 6C). The HBsAg-specific IgG concentration of day 35 in the HBsAg/PPLNP group remained markedly higher in comparison with the HBsAg group (**P < 0.01) or the group adjuvanted with Al (*P < 0.05) (as seen in Fig. 6D). Even on day 42, the IgG concentration of the group adjuvanted with PPLNP stayed markedly elevated when contrasted with the HBsAg set (*P < 0.05) (Fig. 6E). The accelerated and heightened antibody production facilitated by PPLNP underscores its superior efficacy in immune activation.

In mice, IgG2a is associated with T_H_1 responses, commonly characterized by the IFN-γ production and the cytotoxic T cell activation. Conversely, IgG1 is linked to T_H_2 responses, marked by the anti-inflammatory cytokine release and the B cell activation for antibody production. The IgG2a/IgG1 ratio reflects the T_H_1/T_H_2 balance, with a higher ratio indicating a stronger cellular immune response. In our experimental investigation (as seen in Fig. 6F), subsequent to 3 administrations on day 42, the group administered with HBsAg/PPLNP demonstrated notably diminished concentrations of anti-HBsAg IgG1 (associated with T_H_2 response) in comparison with the HBsAg group without adjuvant (*P < 0.05) or the group adjuvanted with Al (*P < 0.05). Conversely, the levels of anti-HBsAg IgG2a (reflecting T_H_1 response) exhibited a marked increase in the HBsAg/PPLNP group when compared with those 2 groups (as seen in Fig. 6G). Additionally, the HBsAg/PPLNP group demonstrated a substantially higher IgG2a/IgG1 ratio than did the HBsAg group without adjuvant (****P < 0.0001) or the group adjuvanted with Al (****P < 0.0001), indicating superior T_H_1 immune stimulation (as seen in Fig. 6H). These results emphasize the heightened efficacy of the PPLNP adjuvant in augmenting T_H_1-driven immune responses, critical for cytotoxic T lymphocyte (CTL) reactions.

### Enlarged IFN-γ secretion, splenocyte proliferation, and cytokine secretion

**Fig. 7.**
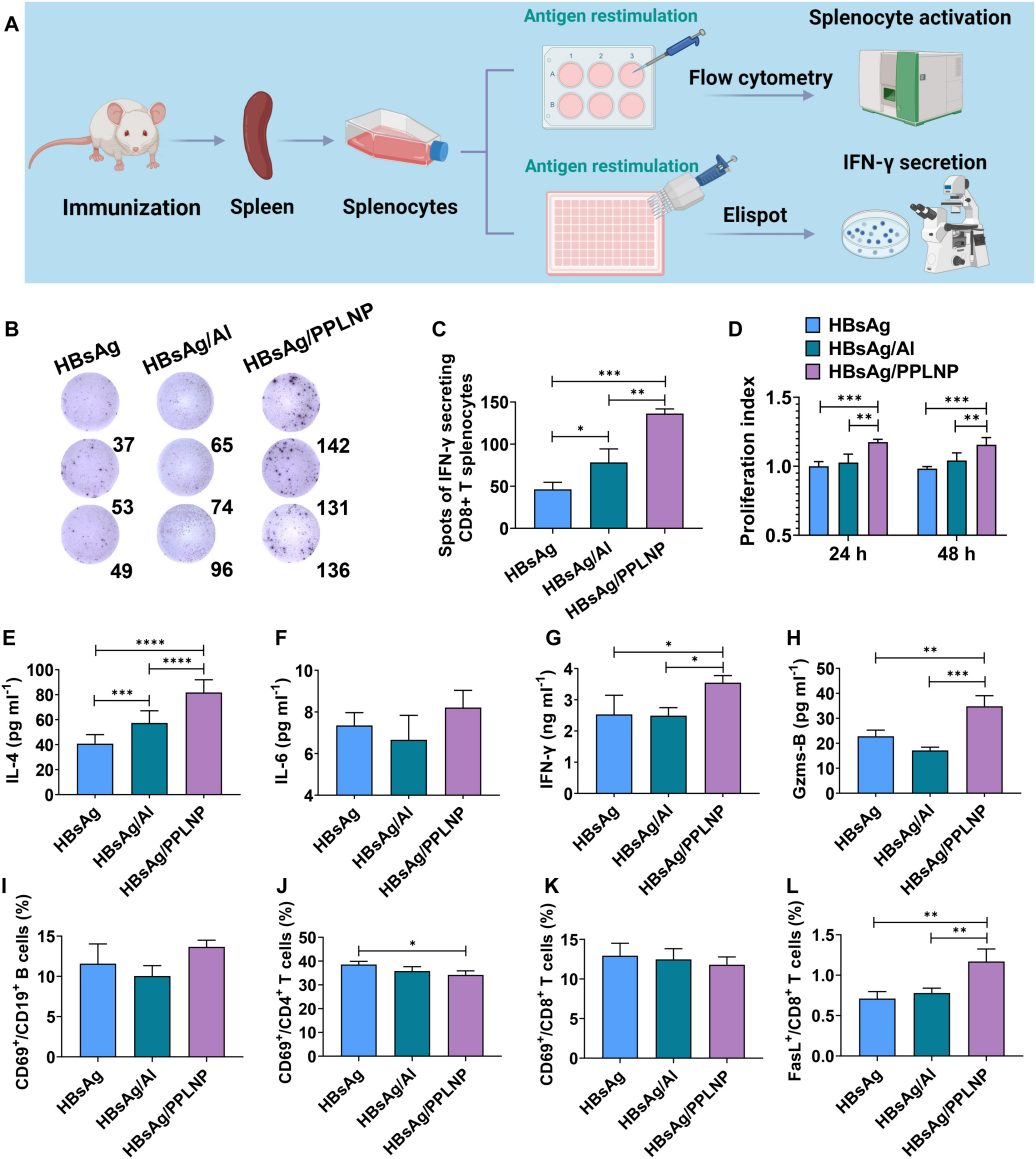
(A) Schematic procedure for analyzing the immunostimulatory effect of the HBsAg/PPLNP nanovaccine in spleen. Created by BioRender. (B) Representative images and (C) quantitative assessment of CD8^+^ T cells producing IFN-γ. (D) Assessment of splenocyte growth after stimulation with HBsAg for 24 or 48 h. (E) Measurement of IL-4, (F) IL-6, (G) IFN-γ, and (H) Gzms-B secretion levels on the day of mouse sacrifice. (I) CD69^+^/CD19^+^ B cells, (J) CD69^+^/CD4^+^ T cells, (K) CD69^+^/CD8^+^ T cells, and (L) FasL^+^/CD8^+^ T cells to characterize splenocyte activation via flow cytometry. *P < 0.05, **P < 0.01, ***P < 0.001, ****P < 0.0001. Statistical analysis was carried out through the use of one-way ANOVA or two-way ANOVA methods.

After second boost of immunization in mice, cytokine secretion and splenocyte activation were assessed (Fig. 7A). Activated T cells are the primary sources of IFN-γ production. Its multifaceted impacts encompass macrophage activation and antiviral defense, augmentation of antigen presentation and generation of MHC–peptide complexes, coordination of lymphocyte–endothelial interactions, modulation of T cell differentiation toward T_H_1/T_H_2 subsets, regulation of cellular proliferation, and facilitation of apoptosis. Following a 48-h restimulation with HBsAg, the HBsAg/PPLNP group exhibited a significantly greater count of CD8^+^ T cells producing IFN-γ in comparison with the HBsAg or HBsAg/Al group, highlighting its cellular immunization trend (as seen in Fig. 7B). Notably, the HBsAg/PPLNP group demonstrated a remarkable 3.0- and 1.7-fold increase in spot numbers than did the HBsAg group (***P < 0.001) and the group adjuvanted with Al (**P < 0.01) (as seen in Fig. 7C). Besides, the proliferation of splenocytes in the HBsAg/PPLNP set was substantially higher compared to the HBsAg set at 24 h (***P < 0.001) and 48 h (***P < 0.001) after being stimulated with the HBsAg antigen (as depicted in Fig. 7D). Similar findings can be found when comparing the HBsAg/PPLNP set to the HBsAg/Al set.

Figure 7E shows that the HBsAg/PPLNP group was of much higher level of IL-4 secretion in comparison with the HBsAg set or the group adjuvanted with Al. IL-4 is well known for its crucial role in regulating T_H_2 immune responses, which includes the differentiation and activation of T_H_2 cells responsible for activating B cells and promoting the antigen-specific antibody secretion. Furthermore, a considerable elevation of IL-6 in the group adjuvanted with PPLNP can be observed when contrasted with the HBsAg or HBsAg/Al groups (as seen in Fig. 7F). IL-6 plays a vital role in antibody production regulation. It exerts influence on B cell activation and differentiation, particularly promoting the following antibody production. Meanwhile, the HBsAg/PPLNP group substantially elevated the IFN-γ level relative to the HBsAg group (*P < 0.05) or the group adjuvanted with Al (*P < 0.05), highlighting their strong T_H_1 driving effect (as seen in Fig. 7G). IFN-γ, which is usually produced by T_H_1 and CD8^+^ T cells, enhances immune function in APCs, promotes T cell activation and proliferation, and regulates T cell subset balance. Additionally, the Gzms-B concentration in the HBsAg/PPLNP group was evidently elevated in comparison with the HBsAg group without adjuvant or the group adjuvanted with Al (as shown in Fig. 7H). Gzms-B is primarily secreted by activated CTLs and natural killer cells. It can trigger apoptosis on targeted cells, eliminating virus-infected cells, and enhance cytotoxic effects of cellular immunity, bolstering antibody-dependent cell-mediated cytotoxicity (ADCC). Compared to aluminum adjuvants, PPLNP significantly enhances splenocyte proliferation and promotes IFN-γ, IL-4, and Gzms-B secretion, thereby exerting a pronounced augmentation of humoral immunity while also exhibiting a more robust trend toward cellular immunity.

### Enhanced activation of splenocytes

Splenocytes are important in the body’s immunization. When splenocytes are activated, they can proliferate and differentiate into various immune cells to support an effective immune defense against pathogens. The activation of splenocytes is vital for triggering and orchestrating the immune response to combat infections and maintain immune homeostasis in the body. No significant difference has been detected in CD69^+^/CD19^+^ B cells within these groups (as seen in Fig. 7I). Interestingly, the proportion of CD69^+^/CD4^+^ T cells in the set adjuvanted with PPLNP was lower than the HBsAg set without adjuvant (Fig. 7J). There were no significant changes observed in CD69^+^/CD8^+^ T cells among these 3 sets (as seen in Fig. 7K). While the CD69^+^/CD8^+^ T cells in the PPLNP-adjuvanted set showed similar results in comparison with the HBsAg group or the group adjuvanted with Al, there was an obvious enhancement in FasL^+^/CD8^+^ T cells compared to both (as seen in Fig. 7L). These results affirmed the significant advantages of the HBsAg/PPLNP group in stimulating cytotoxic T cells. In conclusion, these findings highlighted the exceptional capacity of PPLNP to potentiate the cellular immunity, establishing it as a promising adjuvant for comprehensive immune responses.

### Favorable biocompatibility

**Fig. 8.**
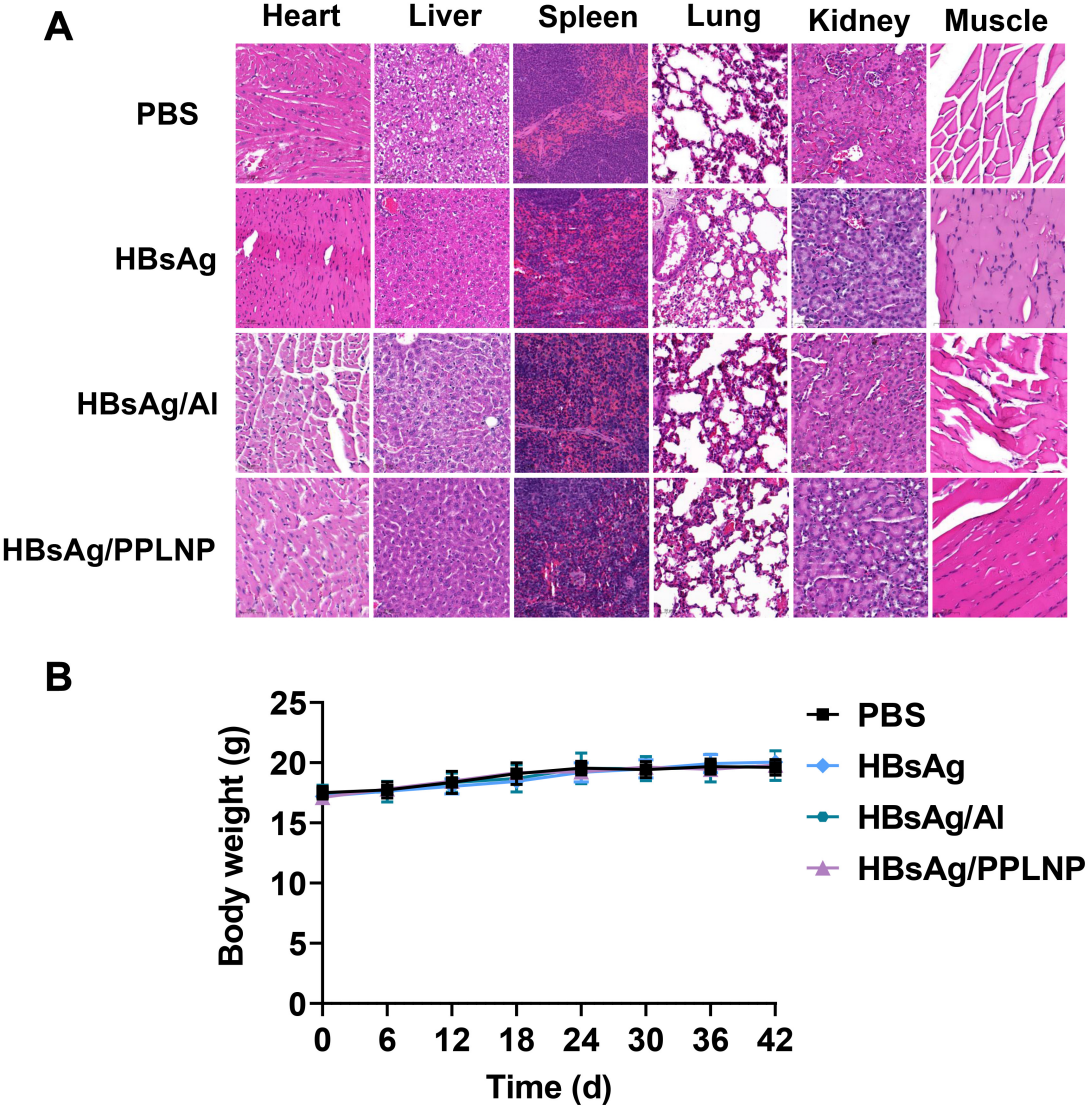
(A) Immunological histochemistry evaluation of diverse organs and the injected portion after primary immunization with distinct vaccine formulations over a 24-h period. (B) Tracking changes in mouse body weights during the course of immunization.

The safety of the HBsAg/PPLNP vaccine was evaluated through immunohistochemical assessments conducted on vital organs and the intramuscular injected portion after initial immunization, with analysis at the 24-h mark (as seen in Fig. 8A). Comparative analysis across the PBS group, as well as the HBsAg, HBsAg/Al, and HBsAg/PPLNP formulations, showed no notable tissue damage in major organs or at the injection site. These results suggest that PPLNPs exhibit favorable biocompatibility and negligible adverse effects. Furthermore, continuous monitoring of the mice’s body weights throughout the immunization process aimed to assess the safety of adjuvants. The minimal fluctuations observed in the HBsAg/PPLNP group further validate the satisfactory biocompatibility associated with the PPLNP adjuvant (as seen in Fig. 8B).

## Discussion

### Enhanced endocytosis and cytoplasmic release of poly I:C

Effective endocytosis followed by efficient cytoplasmic transport of poly I:C is the precondition for RLR activation. It is noteworthy that the majority of rhodamine fluorescence merged with the lysosomal green fluorescence, indicating that most free poly I:C was contained within the endolysosomal system (as seen in Fig. 3B). In contrast, the rhodamine red fluorescence in the PPLNP group diffused throughout the entire cytoplasm, signifying that a substantial portion of poly I:C had breached the lysosomal barrier facilitated by the PPLNP carrier, gaining access to the cytoplasm. This serves as evidence that PPLNP holds great potential for activating intracellular receptors such as RLRs [18]. Besides, the result from flow cytometry suggests an enhanced capability of PPLNP to substantially augment the internalization of poly I:C (as seen in Fig. 3E and F). MHCII molecules attribute to immune responses, primarily facilitating the presentation of exogenous antigenic fragments to CD4^+^ T cells during antigen presentation, thereby activating adaptive immune responses. This proved that PPLNP has advantages in facilitating poly I:C endocytosis and cytoplasmic release.

### RLR activation and type I IFN secretion

The enhanced RLR(RIG-I) activation result (as seen in Fig. 3H) has been proved. This should be attributed to the pronounced lysosomal escape capability of PPLNP, enabling poly I:C to enter the cytoplasm effectively, which has been demonstrated in Fig. 3B. Conversely, the free form of poly I:C lacks the capacity to enter the cytoplasm after entering the lysosome, hence encountering difficulty in activating cytoplasmic RLR. Meanwhile, as type I IFNs serve a key function in boosting cellular immunity and combating viral infections, PPLNP showed advantages in facilitating type I IFN secretion than poly I:C (as seen in Fig. 3I and J), which was consistent with the RLR(RIG-I) activation result.

### Enhanced antigen uptake

Adjuvants often function by adsorbing antigens and generating an antigen reservoir at the injected portion to prolong the interaction between antigens and APCs, thereby enhancing immune responses. The in vitro antigen release results (as seen in Fig. 4A) indicated the potential antigen absorption and depot effect facilitated by PPLNP adjuvant through electrostatic adsorption, thereby enhancing the possibility of sustained antigen presentation and endocytosis by APCs. As free antigens are prone to diffuse at the injected site and induce limited cellular uptake, adjuvants capable of enhancing the antigen endocytosis by APCs are extremely crucial. The stable antigen/adjuvant (HBsAg/PPLNP) formulation formed by the electrostatic absorption between HBsAg and PPLNP offered significant advantages compared to the free antigen (as seen in Fig. 4C to F). First, stabilizing the antigen adsorption onto PPLNP surfaces can reduce the in situ diffusion of antigen. Second, it has been demonstrated that particulate antigens demonstrate higher immunogenicity compared to their soluble counterparts. APCs, including macrophages and DCs, can readily engulf and process particles within the size of 25 nm to 3 μm [33]. Thus, the nanoscale of this HBsAg/PPLNP formulation (~67.8 ± 0.7 nm) favors its uptake by macrophages. Third, this vaccine adjuvant possesses a positive charge, facilitating effective interaction with the APC cell membrane, thereby further enhancing antigen uptake and immunogenicity.

### Antigen depot and lymph node drainage

Prior research has demonstrated that cationic adjuvants exhibit stronger immunogenicity compared to neutral or anionic adjuvants [34,35]. This may be attributed to the association between protein antigens (often negatively charged) and the cationic formulation, which reduces the release of antigen by establishing an antigen reservoir at the injected portion (as seen in Fig. 5B and D). Except for the antigen depot effect, another mechanism attributed to vaccine adjuvants is facilitating the cell-mediated antigen transport to the lymph nodes [36], which has been shown in Fig. 5C and E. This process involves the attraction of migratory immune cells to the injection site from local tissue or the bloodstream. These immune cells are capable of internalizing antigens and subsequently migrating to the draining lymphatic vessels, thereby transporting vaccine components to the draining lymph nodes. This would be attributed from the fact that PPLNP or Al adjuvants decelerate the release of antigen, thereby elongating the antigen migration toward the lymph nodes. Consequently, there is a prolongation in the duration required for the antigens present in the lymph nodes to be degraded or to enter the lymphatic circulation, which provides more opportunities for interaction between APCs and T cells.

In summary, we created a simple poly I:C-incorporated polymer–lipid hybrid adjuvant for the HBsAg vaccine, referred to as PPLNP. The PPLNP synthesis, renowned for its reproducibility, involves a straightforward single-step process, enabling effective encapsulation of poly I:C and facilitating its cellular uptake through self-assembly. Leveraging its “proton sponge effect”, PPLNP assists in the efficient intracellular release of poly I:C, thereby significantly enhancing cytoplasmic RLR (RIG-I) activation, subsequently augmenting IFN-I secretion. Besides, PPLNP notably enhanced antigen uptake by macrophage and extended the antigen’s retention at the injection site, demonstrating an efficient antigen depot effect. Furthermore, PPLNP facilitated antigen drainage to the lymph nodes and reduced antigen clearance in lymph nodes, thereby enhancing interactions between APCs and T cells. As a result, mice immunized with the PPLNP adjuvant exhibited significantly higher IgG concentrations than groups without adjuvant or those adjuvanted with Al even after the second booster. Furthermore, the substantial improvement in the IgG2a/IgG1 ratio within the PPLNP group indicated its capacity to produce a T_H_1-biased immune defense. PPLNP also considerably augmented IFN-γ production, Gzms-B, and FasL^+^/CD8^+^ T cells, surpassing the effects of the commercial Al adjuvant. All these results proved the improved cellular immunity facilitated by PPLNP. Therefore, our novel PPLNP adjuvant exhibits exceptional biocompatibility, facile preparation, cost-effectiveness, and the capability to boost cellular immune responses, making it highly promising for practical applications.

## Data Availability

Data related to the findings in this work can be acquired from the corresponding authors upon reasonable request.
