# Supplementary material for: Enhanced Cellular Immunity for Hepatitis B Virus Vaccine: A Novel Polyinosinic-Polycytidylic Acid-Incorporated Adjuvant Leveraging Cytoplasmic Retinoic Acid-Inducible Gene-Like Receptor Activation and Increased Antigen Uptake
Source: Biomater Res. 2024 Oct 28;28:0096. doi: 10.34133/bmr.0096 (PMC11513446; doi:10.34133/bmr.0096)
Supplement: Supplementary 1 — Figs. S1 and S2 Table S1 [file bmr.0096.f1.docx]

**Enhanced Cellular Immunity for HBV Vaccine: A Novel Poly I:C-incorporated Adjuvant Leveraging Cytoplasmic RLR Activation and Increased Antigen Uptake**

Xuhan Liu^1^, Qiuxia Min^2^, Yihui Li^3*^, Siyuan Chen^4*^

^1^School of Pharmacy, Shenzhen University Medical School, Shenzhen University, No. 1066 Xueyuan Avenue, Shenzhen 518055, China.

^2^Department of Pharmacy, First People’s Hospital of Yunnan Province, Kunming University of Science and Technology, No. 157 Jinbi Road, Kunming, 650034, Yunnan, China.

**** To whom correspondence should be addressed.***

***Yihui Li***

*Email:* *547897750@qq.com*

*^3^ Guangdong Provincial Key Laboratory of Malignant Tumor Epigenetics and Gene Regulation, Guangdong-Hong Kong Joint Laboratory for RNA Medicine, Medical Research Center,* *Sun Yat-Sen Memorial Hospital, Sun Yat-Sen University, Guangzhou 510120, China.*

***Siyuan Chen***

*Email:* *siyuan.chen@njtech.edu.cn*

*^4^Research Institute for Biomaterials, Tech Institute for Advanced Materials, College of Materials Science and Engineering, Suqian Advanced Materials Industry Technology Innovation Center, NJTech-BARTY Joint Research Center for Innovative Medical Technology, Nanjing Tech University, Nanjing, 211816，China*

Table S1. The size distribution, PDI and zeta potential of PPLNP and HBsAg/PPLNP (w/w=1/625) formulation.

| **Sample** | **Size (d. nm)** | **PDI** | **Zeta potential (mV)** |
| --- | --- | --- | --- |
| PPLNP | 83.9±0.3 | 0.27±0.03 | 19.1±2.3 |
| HBsAg/PPLNP (w/w=1/625) | 67.8±0.7 | 0.24±0.01 | 19.4±1.5 |


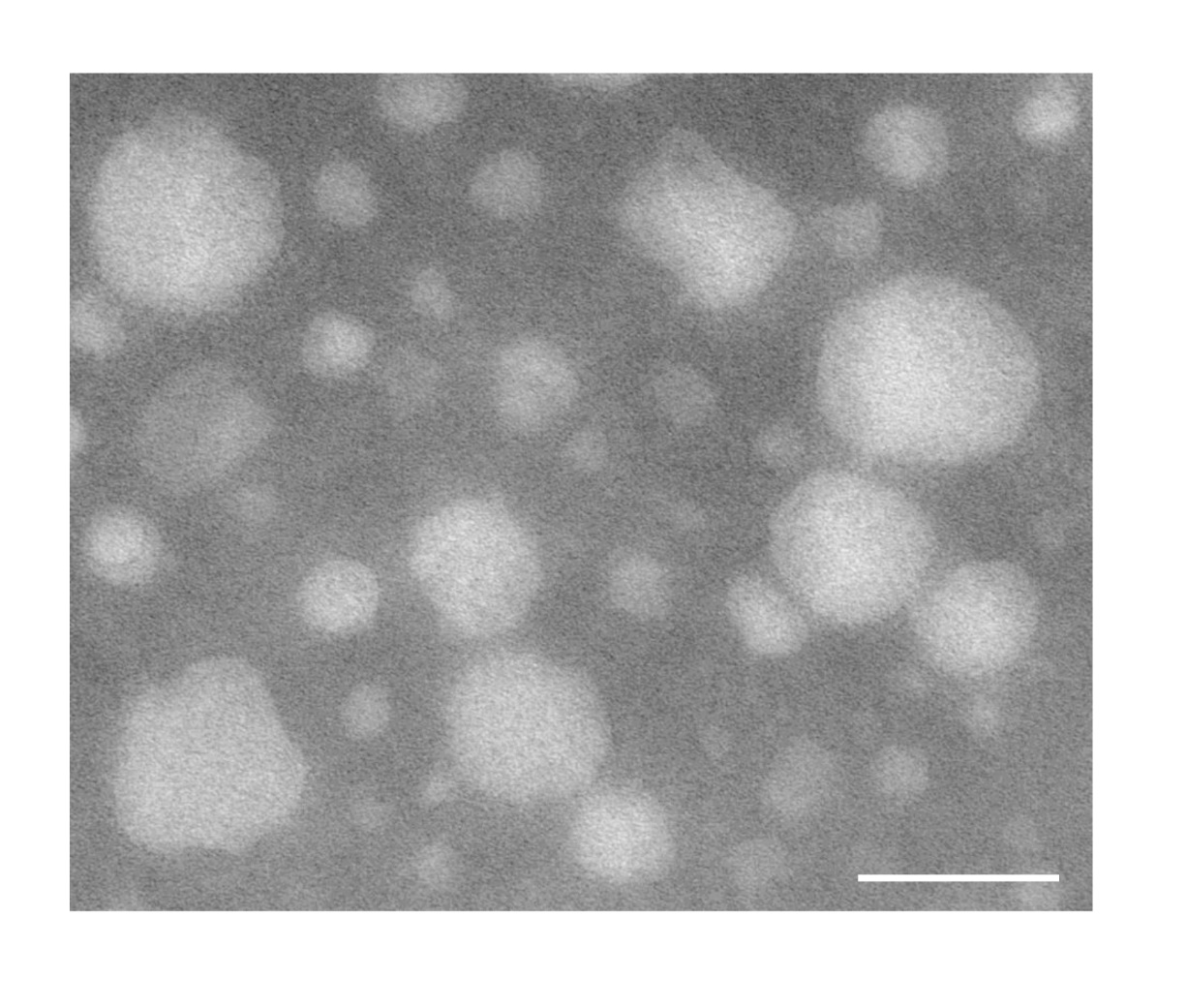


Figure S1. TEM of HBsAg/PPLNP (w/w=1/625) formulation. Bar=50 nm.


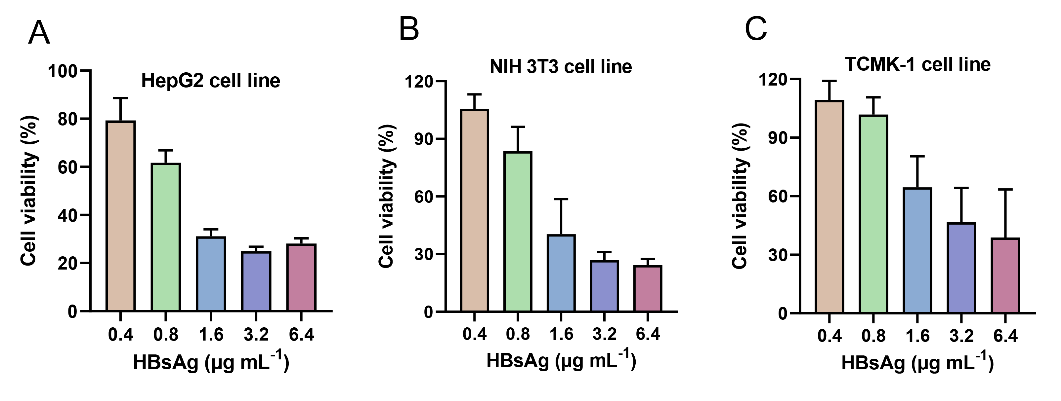


Figure S2. Cell viability of (A) HepG2, (B) NIH 3T3 or (C) TCMK-1 after treatment with different concentrations of HBsAg/PPLNP.
